# Intimate partner violence and help-seeking – a cross-sectional study of women in Sweden

**DOI:** 10.1186/1471-2458-13-866

**Published:** 2013-09-21

**Authors:** Mariana Dufort, Clara Hellner Gumpert, Marlene Stenbacka

**Affiliations:** 1 Department of Clinical Neurosciences, Karolinska Institutet, Stockholm, Sweden; 2 Centre for Psychiatry Research and Education, Stockholm County Council & Karolinska Institutet, Norra stationsgatan 69, plan 7, 113 64 Stockholm, Sweden; 3 Department of Public Health Sciences, Karolinska Institutet, Polhemsgatan 60, plan 1, 112 82 Stockholm, Sweden

## Abstract

**Background:**

Intimate partner violence (IPV) is a global public health concern with possible detrimental consequences for its victims. Studies have found prevalence rates of 15 to 71% for IPV. There is evidence that IPV exposed women perceive barriers to help-seeking and many remain undetected by care givers and authorities. This cross-sectional study aimed to examine IPV exposed women in relation to help-seeking versus non help-seeking from the social services or women’s shelters with regard to social and psychological characteristics as well as relationship with the perpetrator and type of violence exposure.

**Methods:**

Two groups of Swedish IPV exposed women were included: non help-seekers (n = 128) were recruited through ads in newspapers, while help-seekers (n = 347) were recruited from four social service sites and twenty women’s shelters around Sweden. Participants were assessed with questionnaires regarding age, education, occupation and relation to the perpetrator as well as validated instruments measuring psychological distress, psychosocial functioning alcohol use and violence. Analyses were made using Chi^2^ and multivariate logistic regression.

**Results:**

Help-seekers had significantly more often children together with the perpetrator than non help-seekers (64% and 29% respectively) and a high association was found in the fully adjusted model (Adj. OR = 5.46 95% CI 2.99-9.97). Many women in both groups reported a poor social situation and high levels of psychological distress, although more psychological distress was associated with elevated odds for help-seeking (Adj. OR = 2.83 95% CI 1.84-4.34). No differences were found between the groups regarding violence exposure and most women in both groups had experienced severe violence from an intimate partner (95% to 98%).

**Conclusions:**

Results indicate a high problem load among women who had not contacted the social services or women’s shelters due to IPV, and that non help-seekers had similar experiences of severe IPV as help-seekers. This stresses a need to identify IPV exposed women outside specialized settings within the social services and women’s shelters. Asking about partner violence in various health and social care settings could be a feasible strategy to identify battered women and provide them with alternatives for help that ultimately could lead to a life without violence.

## Background

Intimate partner violence (IPV) is an increasingly acknowledged issue both in Sweden and worldwide [1]. According to the World Health Organization (WHO), there is a significant variation in lifetime prevalence of male IPV against women between different countries and regions, ranging from 15% to 71% [2]. Different prevalence estimates are not only due to true differences in the population but also influenced by different data collection methods concerning registration of IPV cases as well as different definitions of IPV [1-4] used in research. In addition, there are inherent measurement problems as IPV mostly occurs in private homes [5,6]. The Swedish National Council for Crime Prevention estimates that 75% of all cases of violence against women in Sweden are unreported to the police [7]. A national study of the Swedish female population [4] showed that a considerable proportion of the violence exposed women had not reported the violence to the police but had instead contacted the health care system or social services.

The detrimental effects of IPV are well documented in both the Swedish and international literature [8-10], with immediate consequences such as injuries, trauma and death [8,11-14] as well as long term impact affecting the victim’s quality of life and health during years after the incidence of violence [11,15]. Long term consequences associated with IPV victimization include poorer mental health and lack of social resources [4,16-18]. In a Canadian study, the adverse consequences of IPV have been shown to increase with violence severity [19]. Still, several studies show that most victims of IPV remain undetected by the health care system [18,20].

### IPV and help-seeking

Even though exposure to IPV involves risks such as injury and trauma, disclosing and/or seeking professional help involves several barriers. Factors such as unemployment, low educational status, economic dependence and experiences of violence have been associated with staying in an abusive relationship [20,21] and also identified as obstacles to disclosure of violence [20,22]. According to American studies, alcohol misuse and the woman’s relationship to the abusive partner can negatively influence her decision to leave or seek help. Alcohol consumption has been identified as a risk factor but also as a consequence of IPV [22]. In addition, violence exposure during childhood has been associated with higher risk of IPV victimization in adulthood [22], as well as with help-seeking patterns [23]. Having children has been contradictory associated with help-seeking. Fear of losing child custody might prevent contacting authorities [20,24], whereas concerns over children’s safety might increase the use of formal support [25]. However, prior research on factors associated with help seeking has mainly been conducted on women who have sought help, or selected groups such as high school or higher education students and ethnic minorities [26].

Variations in help seeking are also influenced by cultural or traditional differences, and attitudes in women’s social environment, including the legal authorities, might play a crucial role in the individual victim’s coping strategy. Studies on national population samples from several countries show that many women turn to informal sources like family and friends before seeking formal support [1,19,27]. Having been badly injured, fearing death or just not being able to endure more were common self-reported reasons for seeking help among IPV exposed women [27,28].

Sweden is known as a welfare state with a broad scope of public services such as local social services and a health care system with an explicit public commitment to ensure equal access to services [29,30]. This has contributed to relatively small inequalities between social classes but also between women and men. Women have free access to maternal health care during pregnancy as well as follow-up health care once the child is born leading to an almost 100% utilization of these services [29,30]. Broader support to families is provided through the community social services. Apart from turning to the police, there are mainly two types of establishments to where women can turn for help due to IPV: non-governmental run women’s shelters or the social services [31]. The high accessibility to different services should make Sweden a relatively low threshold country for help seeking among IPV exposed women. Still, it is estimated that only a minority seek help [7]. Knowledge about social and psychological characteristics of IPV exposed women is essential in order to adequately meet their perceived needs and minimize barriers to help seeking. In the present study, we were able to analyze the social and psychological characteristics of IPV exposed women not seeking help from the social services or women shelters, and compare them with a group of help-seeking women.

### Aim

To study IPV exposed women in relation to help-seeking versus non help-seeking from the social services or women’s shelters with regard to social and psychological characteristics as well as relationship with the perpetrator and type of violence exposure.

Research questions:

Do non help-seeking women differ from the help-seeking women in terms of social and psychological characteristics?

Are there any differences between non help-seeking and help-seeking women regarding relationship to the perpetrator or experiences of violence, including severity and type of violence?

## Methods

### Participants

In the present cross-sectional study data was obtained from two groups of women exposed to IPV by a male partner; non help-seekers (n = 128) and help-seekers (n = 347). Help-seeking was defined as a women’s contact with either a women shelter or the social services due to IPV. Intimate partner violence (IPV) was defined as physical, psychological, or sexual abuse from a present or former sexual/intimate partner. The partner in the woman’s latest violent relationship is referred to as the perpetrator. The study does not include data on whether the women had utilized health care, legal counseling, or informal support from family, friends or other sources.

#### Non help-seeking group

**Figure 1:**
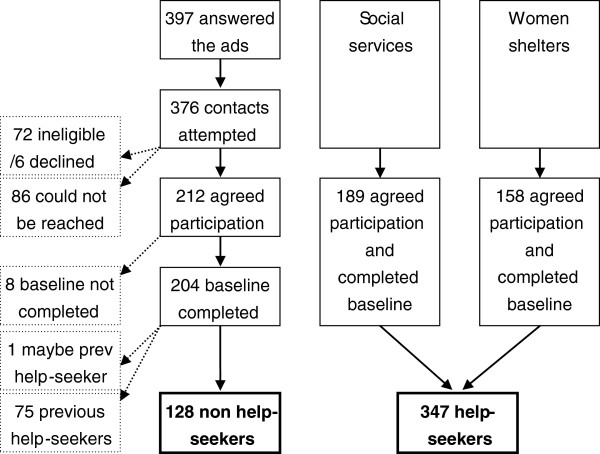
Recruitment of non help-seekers and help-seekers.

Participants in the non help-seeking group were recruited through ads in national and regional daily press and various women’s magazines. The ads were printed at least three consecutive times within a two-week period in each of the newspapers in order to optimize the effect of the ad-out prints. The ad was brief and included a question intended for our target group, and information about a webpage where women could get more information about the study and leave their contact information. Women should be at least 18 years of age and exposed to IPV at least once during the past five years. Furthermore, they should not have been in contact with the social services or a women shelter during the previous year because of IPV. Initially, a total of 397 women were interested in participating and left contact information through the webpage. These were subsequently contacted either by e-mail or by telephone for further information and a short screening to ensure eligibility to the study. Contacts were attempted for 376 women in a consecutive order, and stopped once the desired number of participants was achieved (approximately 200). Of these, 72 women did not meet the inclusion criteria for participation, six repented participation after obtaining further information about the study, and 86 women could not be reached through the contact information they had left via the website. A total of 212 women agreed participation, of which eight did not return the baseline questionnaire. For the purpose of this study, the remaining 204 women were sorted into two groups; those who had been in contact with the social services or women’s shelters at any time (n = 75) and those who had never sought help (n = 128). One woman could not be sorted into any of the groups due to contradictory answers and was excluded. Thus, the final sample of non help-seekers equaled 128 women (Figure 1).

#### Help-seeking group

Recruitment to the help-seeking group was carried out in four community-based social service sites and twenty non-governmental women shelters around Sweden. Women attending these facilities, older than 18 years and with a present experience of violence from an intimate male partner, were asked by the staff at each site if they were interested in obtaining information regarding the present study. Women who were positive to the proposal were contacted by the research staff for further information about the project. As some of these facilities were located in immigrant dense neighborhoods, informed consent and questionnaires were translated to the seven most common languages reported by staff (English, Spanish, Arabic, Persian, Somali, Turkish, Thai and Bosnian). There was some selection bias in the offer to participate, since staff at the included facilities did not inform all help-seeking women about the study. The two most common reasons for not providing information were according to the staff: (1) if the woman’s situation was considered very urgent and (2) if the woman only attended the facility once or did not stay in the shelter for more than a few days. Some women also turned down the offer to participate, and women who did not understand any of the languages available in the survey were excluded. A total of 347 women from the various sites were eventually included in the help-seeking group (Figure 1). Inclusion to the non help-seeking group was carried out from March 2009 to November 2009 and for the help-seeking group from January 2009 to February 2010.

### Procedure

Before participation, all women signed consent information including confidentiality and their right to end participation without need of explanation. The participants were assessed using a set of self-report questionnaires covering psychiatric problems, psychosocial functioning, alcohol use/misuse and exposure to violence. In order to increase safety, women in the non help-seeking group were offered to answer the questionnaire either in a regular postal sent paper format (n = 74) or in a web-survey format (n = 54) sent by mail. Help-seekers completed the paper forms and handed it over to research staff. The Regional Ethical Review Board in Stockholm approved of the study (DNR2008/1269/5).

### Measures

Based on findings from earlier studies on factors associated with IPV [4,19-24,27,28], the following measures on social and psychological characteristics, relationship and violence exposure were included in the study:

*Demographic characteristics*: age (years), place of birth (i.e. if born in Sweden, yes/no), education (Up to high school/University) and occupational status (Part- or fulltime job/Student/Retired/Sick-leave/Unemployed/Long holiday/Other).

*Social life and financial situation*: women’s ability to adapt to and be satisfied with her social role was measured with the Social Adjustment Scale-Self-Report [32] (SAS-SR). SAS-SR includes 54 items addressing specific ways of behaving within a broad range of everyday social contexts, such as interaction with colleagues, neighbors and family. This instrument investigates six social role areas; work role, social and leisure, extended family, primary relationship, parental and family unit. Each item is rated and a mean score is then calculated where higher scores indicate greater impairment. A validation of the total scale has shown good internal consistency (Cronbach’s alpha = 0.74) [33] and good psychometric quality [32]. The social and leisure scale which measures an individual’s relationship with friends and social activities was considered to add most complementary information in relation to other measures added in this study and thus included in the present study. Also, a single item concerning economy was included regarding financial needs.

*Psychological distress*: the presence of psychiatric symptoms and mental health problems was measured with the Symptom Checklist-90-R [34], a 90-item self-report questionnaire designed as a symptom inventory aimed to reflect an individual’s psychological symptom patterns. Each item is rated on a five-point scale of distress (0–4) ranging from “not at all” to “extremely”. The total mean score, Global Severity Index (GSI) which indicates an individual’s level of psychological distress was used in this study. The GSI is the recommended global measure of the Symptom Checklist [34] and it has shown very good internal consistency (Cronbach’s alpha = 0.97) [35]. A Swedish validation study of the instrument suggested a GSI score of 1.21 corresponding to a clinical population and a score of 0.49 to the general population [35].

*Psychosocial functioning*: the Outcome Questionnaire [36] (OQ-30.2) was used, a 30 item self-report questionnaire covering personal and social characteristics that affect an individual’s quality of life. The OQ-30.2 measures psychosocial functioning through three factors; symptom distress, interpersonal relations and social role. A total score measure has been recommended due to high correlation between its subscales [36]. Each item is scored on a five-point likert scale (0–4) and a total score is calculated summing the ratings across all 30 items where a high score indicates more difficulties (Cronbach’s alpha = 0.93). A cut off score of 44 has been recommended to differentiate between normal and reduced psychosocial functioning [36].

*Alcohol use*: alcohol consumption was measured with the Alcohol Use Disorders Identification Test (AUDIT), a 10 item self-report questionnaire developed to screen for excessive drinking [37]. Each item response is scored 0–4 giving a total score between 0 and 40. For women, a score of 6 or above has been recommended as an indication of harmful alcohol use. This test has shown good results in differentiating between normal and problematic alcohol consumption and has been validated in a Swedish context showing very good internal consistency (Cronbach’s alpha = 0.95) [38].

*Relationship to the perpetrator*: single questions about respondents’ current relationship with the perpetrator were included and concerned if the woman remained in the relationship with the perpetrator, if she had children together with him and if she maintained any contact with him (yes/no). Also a question on whether she had a new partner was answered (yes/no).

*Violence*: incidence of violence was measured with the Revised Conflict Tactics Scale (CTS2) [39], a 78-item self-report questionnaire that comprises five scales; negotiation, psychological aggression, physical assault, injury and sexual coercion. Since our focus was on women’s exposure to violence the subscales concerning physical, sexual and psychological violence were used in this study. The CTS2 has been validated in different contexts and is commonly used in research to measure IPV [39,40]. The scales have shown good internal consistency ; with a Cronbach’s alpha of 0.79 to 0.86 for the different scales [39]. For each item respondents are asked to indicate the number of times the violent action has occurred during the past year (never to more than 20 times, or sometime earlier in life). Items can be organized into minor and severe acts [39]. Minor physical violence comprises throwing something, punching, slapping, grabbing or shoving. Severe physical violence includes acts like kicking, biting, hitting with a fist, threatening with a knife or fire gun. Minor psychological violence includes e.g. insults or shouting whereas severe psychological violence refers to threats of violence or destroying partner’s belongings. Minor sexual violence concerns for example insisting on having sex and severe sexual violence includes using threats or force to have sex [39].

The survey included questions about violence exposure (psychological/physical by adults and between parents) during childhood, and violence exposure (psychological, physical and sexual) in previous relationships. The answers were coded dichotomously (yes/no). In addition, women in the non help-seeking group were asked about the last incidence of IPV exposure for each type. Due to contradictory answers between these single questions and the CTS2 on when the violence occurred, violence could not be organized into “happened the past year” or “earlier in life”. Consequently, information about the chronicity according to the CTS2, i.e. frequency of violence during the previous year, could not be calculated.

#### Missing data

Due to the circumstances described above, the number of missing cases was not known and thus there was no possibility to calculate an inclusion rate. The method used to recruit non help-seekers and the lack of information about help-seeking women who declined participation impeded control of missing cases.

The measures were completed by most participants, but some items in the questionnaire were not answered. The internal non-response rates for the specific variables included in the study were 0-8%. Variables *social and leisure*, *psychological distress*, *alcohol use*, *children together with perpetrator*, *physical violence by adults*, *physical violence by adults/between parents*, *any violence during childhood* and *any violence in previous relationships*, had no missing values. One response (0.2%) was missing for variables *psychosocial function* and *physical*, *psychological* and *sexual violence in previous relationships*. Variables *age*, *economy* and *psychological violence by adults during childhood* lacked two responses (0.4%). Three responses (0.6%) were missing concerning *occupation*, four (0.8%) regarding *in a relationship with perpetrator* and six (1.2%) about *education*. Two variables differentiated from the rest with 38 (8%) and 36 (7.5%) missing responses; *new partner* and *maintain contact with perpetrator* respectively. In total, the amount of missing cases in the multivariate analyses did not exceed 10%.

### Statistical analyses

We included the following independent variables in the comparison between the non help-seeking and the help-seeking group: demographics, *social and leisure*, *psychological distress*, *psychosocial functioning*, *alcohol use*, *violence during childhood*, *violence in previous relationships*, relationship status, *children together with the perpetrator* and violence conducted by the perpetrator (Tables 1, 2 and 3). *Help-seeking* (yes/no) was used as the dependent variable/outcome measure. A correlation matrix with binary statistics between all variables using Spearman’s Rank Order correlation was initially conducted to get an overview of the data before further analyses. In order to control for possible independent group differences, the chi-square test of independence and the Mann–Whitney U test for variables with nonparametric score distributions were used. Prior to the multivariate analysis, linearity among the continuous variables was checked for by grouping them into categories. This procedure was followed in order to avoid false assumptions of linear correlations between continuous independent variables and the outcome (help-seeking). All continuous variables in the model showed a linear association with help-seeking and were thus included in the analyses.

**Table 1:** Non help-seeking and help-seeking women’s psychosocial characteristics and relationship status (n = 475)

**Variable**	**Non-help-seekers**	**Help-seekers**	**Sign**
	**(n=128)**	**(n=347)**	
	**%/Mean (SD)**	**%/Mean (SD)**	**(Chi**^**2**^**)**
Age (years)	40.1 (11.71)	34.1 (9.90)	.000^a^
Foreign born			.000
Born in Sweden	86%	49%	
Born outside Sweden	14%	51%	
Education			.000
Upper secondary school	47%	72%	
University	53%	28%	
Occupation			.000
Part- or fulltime job	49%	32%	
Student	10%	21%	
Retired/sick-leave	22%	13%	
Unemployed/long holiday	12%	29%	
Other	7%	5%	
Economy			.009
Enough money for needs	50%	32%	
Usually enough money	11%	16%	
Not enough, did not had to borrow	15%	17%	
Not enough, had to borrow	12%	14%	
Great financial difficulty	12%	21%	
Social and leisure (SAS)	2.2 (0.62)	2.6 (0.69)	.000^a^
Psychological distress (GSI)	1.1 (0.74)	1.5 (0.76)	.000^a^
Psychosocial function (OQ)	45.7 (20.95)	55.7 (18.13)	.000^a^
Alcohol use/misuse (AUDIT)	5.1 (5.60)	3.0 (4.91)	.000^a^
***Relationship status***			
In a relationship with perpetrator	10%	23%	.003
Children together with perpetrator	29%	64%	.000
Maintain contact with perpetrator	39%	39%	.983
New partner	36%	10%	.000

^a^*Group differences were tested using Mann–Whitney U Test.*

**Table 2:** Experiences of violence among non help-seekers and help-seekers (n = 475)

**Variable**	**Non-help-seekers**	**Help-seekers**	**p-value**
	**(n=128)**	**(n=347)**	**(Chi**^**2**^**)**
***Violence during childhood***			
Psychological by adults	52%	52%	.908
Physical by adults	48%	46%	.722
Psychological between parents	47%	47%	.987
Physical between parents	28%	33%	.325
Any violence during childhood	67%	68%	.818
***Violence in previous intimate relationships***			
Psychological	53%	46%	.181
Physical	42%	34%	.104
Sexual	20%	19%	.817
Any	61%	50%	.036
***Violence in latest violent relationship***			
Minor psychological	100%	100%	.543
Severe psychological	88%	95%	.004
Minor physical	95%	97%	.004
Severe physical	89%	91%	.575
Minor sexual	89%	69%	.189
Severe sexual	38%	43%	.332
Any severe	95%	98%	.080
Last incidence of violence			.000
During the last year	35%	91%	
Earlier in life	65%	9%	

**Table 3:** Women’s characteristics by help-seeking versus non help-seeking (n = 428)

**Variable**	**Help-seeking**	
	**Adj. OR (95% CI)**	**p-value**
Age (years)	0.94 (0.91–0.96)	.000
Education		
Up to high school	1	
University	0.38 (0.22–0.67)	.001
Psychological distress (GSI)	2.83 (1.87–4.29)	.000
Alcohol consumption (AUDIT)	0.94 (0.89–0.99)	.029
In a relationship with the perpetrator		
No	1	
Yes	3.92 (1.24–12.40)	.020
Children together with the perpetrator		
No	1	
Yes	5.46 (2.99–9.97)	.000
New partner		
No	1	
Yes	0.19 (0.09–0.37)	.000

Hosmer-Lemeshow = 0.19.

Nagelkerke R square = 0.49.

Only statistically significant variables from the binary tests, along with significant correlation variables in the initial correlation matrix were included in the multivariate analysis. Due to multicollinearity between the variables *social and leisure*, *psychosocial functioning* and *psychological distress*, only the latter variable (*psychological distress*) was kept in the multivariate analyses as it was deemed of greatest value to report. Multivariate logistic regression models with a backward stepwise approach were conducted to investigate potential differences between the groups, thus results are presented as adjusted odds ratios (Adj. OR) with 95% confidence interval (CI). The goodness-of-fit of the multivariate models were tested with the Hosmer-Lemeshow test and Nagelkerke R square [41]. A listwise deletion approach was used to deal with missing data, meaning that only complete cases were included in the multivariate analyses. Analyses of missing data did not show any significant differences between those who responded to all items and those who did not, thus this approach was chosen since it facilitates comparability as all calculations proceed from a common base [42]. Given the exclusion of missing cases the final sample size in the multivariate logistic regression model included 428 cases i.e. 90% of the initial sample of 475 women and the proportion of included cases were similarly distributed by group (114 non help-seekers and 314 help-seekers). All statistical analyses were performed using the statistical program package SPSS, version 20.0.

Two variables could not be included in the multivariate analysis due their interaction with other independent variables in combination with few observations in the non help-seeking group. There were too few non help-seekers born outside Sweden or with IPV exposure during the last year. Only the group of women born in Sweden was possible to analyze separately in a multivariate logistic model. However, the variables *foreign born* and *last incidence of violence* were separately analyzed together with independent variables in our final model. Separate independent sample t-tests for help-seeking with split file by *foreign born* were conducted on the continuous independent variables *age*, *psychological distress* and *alcohol use*. Analyses with discrete variables; *education*, if still *in a relationship with the perpetrator*, if *children together with the perpetrator* and if she has a *new partner*, were conducted using cross-tabulations and Chi^2^ with split file by *foreign born*. The same procedure was carried out when examining the variable *last incidence of violence* coded as “during the last year” or “earlier in life” i.e. cross-tabulations with Chi^2^ and independent sample t-tests with split file by last incidence of violence.

## Results

### Women’s social and psychological characteristics

As presented in Table 1, women in the non help-seeking group were on average 6 years older and more often born in Sweden as compared to help-seekers. More than two thirds of the help-seekers had upper secondary school, while more than half of non help-seekers had university education. It was more common among non help-seekers to have a part- or fulltime job or be retired/in sick leave whereas help-seekers were more often students or unemployed. Furthermore, more women in the non help-seeking group reported having sufficient economy according to their needs than help-seekers (50% vs. 32%).

Women in the non-help seeking group had an average score of 2.2 on the *Social and leisure* scale, which indicates a poor social life, while the help-seeking women reported an even poorer social life (score = 2.6). Most women in both groups reported high psychological distress and low psychosocial functioning, although non help-seeking women reported lower levels of psychological distress and a higher psychosocial function compared to help-seekers. Also, non help-seekers reported higher alcohol consumption (score = 5.1) than did help-seekers (score = 3.0).

### Relationship with perpetrator and experiences of violence

Non help-seekers less often had an ongoing relationship (10% vs. 23%) or had children together with the perpetrator than did help-seekers (29% vs. 64%), and more women in the non help-seeking group (36% vs. 10%) had a new partner (Table 1).

Table 2 displays women’s experiences of violence, type of violence and help-seeking. The majority of the women in both groups (67% and 68%) had been exposed to violence during their childhood; psychological violence was most prevalent and no differences concerning childhood violence experiences were found between the groups. There was a difference in the overall prevalence of violence in prior relationships between non help-seekers and help-seekers (61% and 50% respectively) but no differences in prevalence per type of violence.

The majority of women in both groups (95% and 98%) were exposed to some kind of severe violence in their latest violent relationship. Severe psychological violence was less prevalent among non help-seekers than help-seekers (88% vs. 95%, p = .004). Most women in the non help-seeking group had their last incidence of violence earlier in life whereas the vast majority of help-seekers had experienced partner violence during the previous year. No other differences were found between the groups concerning IPV experiences.

### Women’s characteristics and help-seeking

In the multivariate analyses, characteristics concerning *age*, *education*, level of *psychological distress*, *alcohol use*, remaining *in the relationship with the perpetrator*, *children together with the perpetrator* and having a *new partner*, differed between non help-seekers and help-seekers (Table 3). Older age (Adj. OR = 0.94, 95% CI 0.91-0.96) and having a higher education (Adj. OR = 0.38 95% CI 0.22-0.67) was associated with lower likelihood of seeking help. In contrast, higher levels of psychological distress were associated (Adj. OR = 0.83 95% CI 1.87-4.29) with help-seeking in multivariate analyses. Higher alcohol consumption was related to lower odds of help-seeking (Adj. OR = 0.94 95% CI 0.89-0.99). Remaining in the relationship with the perpetrator was associated with nearly fourfold increased likelihood of seeking help (Adj. OR = 3.92 95% CI 1.24-12.40). Women who had children together with the perpetrator more than fivefold higher odds to seek help (Adj. OR = 5.46 95% CI 2.99-9.97). Having a new partner was associated with a decreased likelihood of help-seeking (Adj. OR = 0.19 95% CI 0.09-0.37).

When analyzing *foreign born*, younger age and higher levels of psychological distress were associated with help-seeking among Swedish born women but not among women born abroad. The association between alcohol use and lower likelihood of help-seeking was only true for women born outside Sweden. All other associations in both groups remained the same as in the logistic model. The multivariate analyses of the Swedish born women confirmed these results. Considering women’s last incidence of IPV exposure showed that among women exposed earlier in life, only *age*, levels of *psychological distress* and having *children together with the perpetrator* were associated with help-seeking. Further, remaining in a relationship with the perpetrator or having a new partner was not associated with help-seeking among women exposed to IPV during the last year (results not shown in any table).

## Discussion

### Main findings

The overall aim of this study was to explore social and psychological factors related to help-seeking among women exposed do IPV. A main finding was that the vast majority of both help- and non help-seeking women had experienced severe violence in their latest violent relationship; no significant differences between non help-seekers and help-seekers were found. The most common type of violence exposure was psychological followed by physical and sexual violence.

A second finding was that non help-seekers were on average older, had higher education and better social and psychological conditions compared to help-seekers which entails that this group has probably more recourses considering social network, family and other supportive significant others. The results showed differences in women’s psychological health and socioeconomic status in favor for non-help seekers, while no differences were found between non help-seekers and help-seekers concerning experiences of violence.

Another important finding was that women in both groups reported high levels of psychological distress although help-seekers suffered from more severe psychological and social problems. Differences in psychological distress might partly be due to differences in time to the most recent incidence of violence, where help-seekers experienced violence more recently than did non help-seekers. Previous research has suggested that many women are confronted with a range of challenges upon leaving the violent relationship, making them more vulnerable to increased violence exposure and poorer psychological health [43-45]. In this study, we found an association between help-seeking and higher psychological distress despite last incidence of violence. This would conversely indicate long-term consequences of the violent experiences stressing a need of support regardless of when the last IPV experience had occurred.

### Social and psychological characteristics

Consistent with previous research showing a negative association between alcohol consumption and help-seeking due to IPV (Table 3) [22,46], non help-seekers in this study reported higher alcohol consumption than help-seekers. However, separate analyses by *foreign born* showed no association between alcohol consumption and help-seeking among Swedish born women. The association between higher alcohol consumption and non help-seeking was only valid among women born outside Sweden. Similar findings have been reported in a Norwegian study of help-seeking IPV exposed women where foreign born women reported less alcohol consumption than native born help-seeking women [47]. One possible explanation could be that foreign born women who consume alcohol are more reluctant to seek formal support than their native counterparts, or that alcohol use is associated to greater feelings of shame among foreign born than among native born women in Scandinavia.

In line with earlier research [25,28,45], having children together with the perpetrator was strongly associated with help-seeking. In contrary to findings where fear of losing custody of the children might prevent help-seeking [20,24], women in the help-seeking group were more likely to have children compared to non help-seekers. Our results might indicate that custody disputes are not critical thresholds for help-seeking in Sweden maybe due to high accessibility of family social support and maternal care [30]. One explanation could be the high access to maternal and child care with an almost 100% attendance to these services among women in Sweden. This might help detection of IPV cases but also increase women’s confidence in help-seeking.

### Violence exposure

In contrast to other studies, e.g. Popescu and colleagues [23], we found no differences regarding exposure to childhood violence between the groups, and the majority reported such experiences. Consistent with previous research on the risk of repeated violence [48], most women in both groups had also experienced some kind of violence in a previous intimate relationship. Since the majority of women had been exposed to repeated violence, the fact that they reported high degrees of psychological distress and poor psychosocial functioning may not be surprising. In this aspect, our findings are in line with earlier studies showing an association between repeated victimization and poorer psychological health [49-52].

### Help-seeking

As social support has been shown to reduce the adverse consequences of IPV on battered women's quality of life and health consequences [53-57], it is important to further work to minimize or eliminate barriers to help-seeking. This study did not focus on such barriers in particular but instead possible differences related to help-seeking and non help-seeking. The results indicate a high problem load among women who had not contacted social services or women shelters, but we have no data indicating whether these women might utilize other services for support, e.g. somatic and/or mental health care. Families’ high access to a broad spectrum of social support through local community services way increase the chance for the social services to come in contact with non help-seeking battered women for other reasons than IPV. Results from bivariate analyses in this study indicate that non help-seekers have similar experiences of severe IPV as help-seekers but tend to have a better financial situation compared to help-seekers. This stresses the need to identify these women outside the specialized departments for battered women within the social services. Another possible way to reach out to those in need could be to target various health care settings – in particular those providing maternal health care, which reach the majority of women in Sweden- with general information of partner violence and its consequences, where to seek help etc. could be implemented as a strategy to reach out to battered women [58]. Midwifes and nurses could be trained to ask questions about partner violence, which could be trigger for both disclosures as well as providing opportunities to offer concrete help tapping in to the individual needs of the woman and her possible children [59].

### Strengths and limitations

One limitation of the study is that we only asked for children together with the perpetrator and not if other children existed in the relationship, which may have lead to a possible underestimation of the importance of children for help-seeking. Another limitation is the lack of information about women in shelters and social service who declined participation or were not invited to participate; hence it was not possible to characterize missing cases in relation to responders. Also, by using a listwise deletion approach missing data on the variable “New partner” reduced our sample by almost 10% in the final model. This may have primarily influenced the results concerning variables “In a relationship with the perpetrator” and “Children together with the perpetrator” which could have a confounding association to “New partner” and help-seeking. On the other hand, no correlation variables remained significant in the final model indicating that the influence of missing data on our results are considered small. In addition, some differences between the groups might be better explained by selection bias rather than help-seeking/non help-seeking. Non help-seekers, who were recruited through ads, were to a greater extent born in Sweden compared to help-seekers, and help-seekers were to some extent recruited in sites located in immigrant dense neighborhoods. Also, the prevalence of last episode of violence differed between the groups, though non help-seekers were included if they have had an IPV experience sometime during the past five years whereas most help-seekers turned to the sites due to present IPV. These differences decreased the accuracy of group comparisons in relation to help-seeking because this variable could not be included in the multivariate analysis (Table 3). On the other hand, recruiting non help-seeking battered women through ads contribute with information about a group of women of whom we know relatively little about.

The present study adds knowledge about non help-seeking women who have not been recruited from hospitals or correctional authorities and could be considered as a more general group of women with experience of intimate partner violence. These women may represent a group with relatively strong recourses why many of them have managed to end their violent situation without specialized formal help. The study also contributes with new knowledge about non help-seeking women and social characteristics that could be related to help-seeking in the context of a country with a well developed social welfare system [29,30].

For the future, ethnical differences should be further studied in terms of societal inequalities extending our knowledge of immigrants’ specific vulnerable situation compared to natives in the context of Western countries. Also, prospective longitudinal studies of help-seekers and non help-seekers would substantially increase our understanding of internal and contextual conditions that may influence decision making with regard to help-seeking.

## Conclusion

Help-seeking women suffered from more psychological distress indicating a greater need of formal help compared to non help-seekers. However, also non help-seeking women reported a poor social situation as well as high levels of psychological distress and had similar substantial lifetime experiences of violence as help-seekers. Results suggest a need to identify IPV exposed women outside specialized settings within the social services and women’s shelters. Asking about partner violence in various health and social care settings might be a possible strategy to identify battered women and provide them with alternatives for help that ultimately could lead to a life without violence.

## Competing interests

The authors declare that they have no competing interests.

## Authors’ contributions

MD participated in the design of the study, carried out the data collection, analyzed the data and drafted the manuscript. CHG helped draft the manuscript. MS participated in the design of the study, advised on analysis of results and helped draft the manuscript. All authors read and approved the final manuscript.

## Pre-publication history

The pre-publication history for this paper can be accessed here:

http://www.biomedcentral.com/1471-2458/13/866/prepub

## References

[B1] Garcia-Moreno C Jansen HAFM Ellsberg M Heise L Watts C WHO multi-country study on women’s health and domestic violence against women. Initial results on prevalence, health outcomes and women’s responses Book WHO multi-country study on women’s health and domestic violence against women. Initial results on prevalence, health outcomes and women’s responses 2005 Geneve: WHO World Health Organization

[B2] Garcia-Moreno C Prevalence of intimate partner violence: findings from the WHO multi-country study on women’s health and domestic violence Lancet 2006 368 1260 1269 10.1016/S0140-6736(06)69523-8 17027732

[B3] Coker A Smith PH McKeown RE King MJ Frequency and correlates of intimate partner violence by type: physical, sexual, and psychological battering Am J Public Health 2000 90 553 559 10754969 10.2105/ajph.90.4.553 PMC1446198

[B4] Lundgren E Heimer G Westerstrand J Kalliokoski A-M Slagen dam 2001 Stockholm, Sweden: Fritzes

[B5] Straus M Manual for the conflict tactics scale Book manual for the conflict tactics scale 1990 Durham: Family Research Laboratory

[B6] Krug EG Dahlberg L Mercy JA Zwi AB Lozano R World report on violence and health, world report on violence and health 2002 Geneve: World Health Organization

[B7] Brottsförebyggande rådet Brottsutvecklingen i Sverige fram till år 2007 2008 Stockholm: Brottsförebyggande rådet vol. 2008:23

[B8] Golding JM Intimate partner violence as a risk factor for mental disorders: a meta-analysis J Fam Violence 1999 14 99 132 10.1023/A:1022079418229

[B9] Vos T Astbury J Piers LS Magnus A Heenan M Stanley L Walker L Webster K Measuring the impact of intimate partner violence on the health of women in Victoria, Australia World Health Organization 2006 84 739 744 10.2471/BLT.06.030411 PMC2627471 17128344

[B10] Leander K Mäns våld mot kvinnor: Ett folkhälsoproblem (violence of men against women: a public health problem) Book mäns våld mot kvinnor: Ett folkhälsoproblem (violence of men against women: A public health problem) 2007 Stockholm: Karolinska Institutet School of Public Health

[B11] Campbell J Intimate partner violence and physical health consequences Archives of internal medicine 2002 162 1157 1163 10.1001/archinte.162.10.1157 12020187

[B12] Campbell JCC Health consequences of intimate partner violence Lancet 2002 359 1331 1336 10.1016/S0140-6736(02)08336-8 11965295

[B13] Walker LEA Battered woman syndrome Ann NY Acad Sci 2006 1087 142 157 10.1196/annals.1385.023 17189503

[B14] Kaura SA Lohman BJ Dating violence victimization, relationship satisfaction, mental health problems, and acceptability of violence: a comparison of men and women J Fam Violence 2007 22 367 381

[B15] Zlotnick C Intimate partner violence and long-term psychosocial functioning in a national sample of american women Journal of interpersonal violence 2006 21 262 275 10.1177/0886260505282564 16368765

[B16] Bensley L Van Eenwyk J Wynkoop Simmons K Childhood family violence history and women’s risk for intimate partner violence and poor health American journal of preventive medicine 2003 25 38 44 12818308 10.1016/s0749-3797(03)00094-1

[B17] Teplin LA McClelland GM Abram KM Weiner DA Crime victimization in adults with severe mental illness: comparison with the national crime victimization survey Arch Gen Psychiatry 2005 62 911 921 10.1001/archpsyc.62.8.911 16061769 PMC1389236

[B18] Howard LM Trevillion K Khalifeh H Woodall A Agnew-Davies R Feder G Domestic violence and severe psychiatric disorders: prevalence and interventions Psychol Med 2010 40 881 893 10.1017/S0033291709991589 19891808

[B19] Ansara DL Hindin MJ Formal and informal help-seeking associated with women’s and men’s experiences of intimate partner violence in Canada Soc Sci Med 2010 70 1011 1018 10.1016/j.socscimed.2009.12.009 20122774

[B20] Plichta SB Falik M Prevalence of violence and its implications for women’s health Womens Health Issues 2001 11 244 258 10.1016/S1049-3867(01)00085-8 11336864

[B21] Barnett OW Why battered women do not leave, part 1: external inhibiting factors within society Trauma Violence Abuse 2000 1 343 372 10.1177/1524838000001004003

[B22] Hien D Ruglass L Interpersonal partner violence and women in the United States: an overview of prevalence rates, psychiatric correlates and consequences and barriers to help seeking Int J Law Psychiatry 2009 32 48 55 10.1016/j.ijlp.2008.11.003 19101036 PMC3468326

[B23] Popescu ML Drumm R Dewan S Rusu C Childhood victimization and its impact on coping behaviors for victims of intimate partner violence J Fam Violence 2010 25 575 585 10.1007/s10896-010-9317-5

[B24] Wolf ME Ly U Hobart MA Kernic MA Barriers to seeking police help for intimate partner violence J Fam Violence 2003 18 121 129 10.1023/A:1022893231951

[B25] Fugate M Landis L Riordan K Naureckas S Engel B Barriers to domestic violence help seeking: implications for intervention Violence against women 2005 11 290 310 10.1177/1077801204271959 16043551

[B26] Nerøien AI Schei B Partner violence and health: results from the first national study on violence against women in Norway Scand J Public Health 2008 36 161 168 10.1177/1403494807085188 18519280

[B27] Barrett BJ Pierre MS Variations in Women’s help seeking in response to intimate partner violence: findings from a Canadian population-based study Violence against women 2011 17 47 70 10.1177/1077801210394273 21199809

[B28] Fanslow JL Robinson EM Help-seeking behaviors and reasons for help seeking reported by a representative sample of women victims of intimate partner violence in New Zealand Journal of interpersonal violence 2010 25 929 951 10.1177/0886260509336963 19597160

[B29] Lundberg O Åberg Yngwe M Stjärne M Björk L Fritzell J The nordic experience: welfare states and public health (NEWS): in book the nordic experience: welfare states and public health (NEWS) 2008 Stockholm: City

[B30] Anell A Glenngård AH Merkur S Sweden: health system review Book Sweden: Health system in Transition 2012 14 5 1 159 22894859

[B31] Antilla S Ericson C Glad J Fredriksson M Olofsson H Smedslund G Utfall och effekter av sociala metoder för kvinnor som utsatts för våld i nära relationer: En systematisk översikt Book utfall och effekter av sociala metoder för kvinnor som utsatts för våld i nära relationer: en systematisk översikt 2006 Stockholm: Socialstyrelsen

[B32] Weissman MM Social adjustment scale-self-report (SAS-SR): technical manual Book social adjustment scale-self-report (SAS-SR): technical manual 1999 New York:

[B33] Edwards DW Yarvis RM Mueller DP Zingale HC Wagman WJ Test-taking and the stability of adjustment scales: can we assess patient deterioration? Eval Rev 1978 2 275 291 10.1177/0193841X7800200206

[B34] Derogatis LR SCL-90-R: symptom checklist-90-revised: administration, scoring, and procedures manual Book SCL-90-R: symptom checklist-90-revised: administration, scoring, and procedures manual 1994 Minneapolis:

[B35] Fridell M SCL 90: svensk normering, standadisering och validering av symtomskalan Book SCL 90: svensk normering, standadisering och validering av symtomskalan 2002 Stockholm: Statens Institutionsstyrelse

[B36] Lambert MJ Finch MA Okiishi J Burlingame GM Administration and scoring manual for the OQ-30.2: a brief outcome and tracking questionnaire for adults 2005 Salt Lake City, UT: OQ Measures, LLC

[B37] Saunders JB Aasland OG Babor TF De La Fuente JR Grant M Development of the alcohol use disorders identification test (AUDIT): WHO collaborative project on early detection of persons with harmful alcohol consumption-II Addiction 1993 88 791 804 10.1111/j.1360-0443.1993.tb02093.x 8329970

[B38] Bergman H Källmén H Alcohol use among swedes and a psychometric evaluation of the alcohol Use disorders identification test Alcohol Alcohol 2002 37 245 251 10.1093/alcalc/37.3.245 12003912

[B39] Straus MA Hamby SL Warren LW The conflict tactics scales handbook 2003 Los Angeles, CA: Western Psychological Services

[B40] Straus M Hamby SL Boney-Mc Coy S Sugarman D The revised conflict tactics scale (CTS2): development and preliminary psychometric data J Fam Issues 1996 17 283 316 10.1177/019251396017003001

[B41] Vittinghoff E Glidden DV Shiboski SC McCulloch CE Regression methods in biostatistics linear, logistic, survival, and repeated measures models 2012 2 New York: Springer 509 Statistics for Biology and Health

[B42] Little RJA Rubin DB Statistical analysis with missing data 2002 2 New Jersey: John Wiley & Sons

[B43] Anderson DK Saunders DG Leaving an abusive partner Trauma Violence Abuse 2003 4 163 191 10.1177/1524838002250769 14697121

[B44] Ekbrand H Separationer och mäns våld mot kvinnor 2006 Doctoral thesis: University of Gothenburg, Department of Sociology

[B45] Moe AM Battered women, children, and the end of abusive relationships Affilia 2009 24 244 256 10.1177/0886109909337374

[B46] Lipsky S Caetano R Field CA Larkin GL The role of intimate partner violence, race, and ethnicity in help-seeking behaviors Ethn Health 2006 11 81 100 10.1080/13557850500391410 16338756

[B47] Vatnar SKB Bjorkly S An interactional perspective on the relationship of immigration to intimate partner violence in a representative sample of help-seeking women Journal of interpersonal violence 2010 25 1815 1835 10.1177/0886260509354511 20040712

[B48] Kuijpers KF Van der Knaap LM Winkel FW Victims’ influence on intimate partner violence revictimization : an empirical test of dynamic victim-related risk factors Journal of interpersonal violence 2011 12 198 219 10.1177/0886260511430389 22203626

[B49] Areta CM From child victim to adult victim: a model for predicting sexual revictimization Child Maltreat 2000 5 28 38 10.1177/1077559500005001004 11232060

[B50] Banyard VL Williams LM Siegel JA The long-term mental health consequences of child sexual abuse: an exploratory study of the impact of multiple traumas in a sample of women J Trauma Stress 2001 14 697 715 10.1023/A:1013085904337 11776418

[B51] Fogarty CT Fredman L Heeren TC Liebschutz J Synergistic effects of child abuse and intimate partner violence on depressive symptoms in women Prev Med 2008 46 463 469 10.1016/j.ypmed.2007.12.009 18207563

[B52] Scott-Storey K Cumulative abuse: do things add up? an evaluation of the conceptualization, operationalization, and methodological approaches in the study of the phenomenon of cumulative abuse Trauma Violence Abuse 2011 12 135 150 10.1177/1524838011404253 21511684

[B53] Coker AL Physical and mental health effects of intimate partner violence for men and women American journal of preventive medicine 2002 23 260 268 10.1016/S0749-3797(02)00514-7 12406480

[B54] Coker A Social support reduces the impact of partner violence on health: application of structural equation models Preventive medicine 2003 37 259 267 10.1016/S0091-7435(03)00122-1 12914832

[B55] Goodkind JR Gillum TL Bybee DI Sullivan CM The impact of family and friends’ reactions on the well-being of women with abusive partners Violence against women 2003 9 347 373 10.1177/1077801202250083

[B56] Bauman EM Social support and loss of resources as predictors of mental health and quality of life in battered women over time. Doctoral thesis 2009 American University, Department of Psychology

[B57] Postmus JL Severson M Berry M Yoo JA Women’s Experiences of violence and seeking help Violence against women 2009 15 852 868 10.1177/1077801209334445 19458091

[B58] Selic P Pesjak K Kersnik J The prevalence of exposure to domestic violence and the factors associated with co-occurrence of psychological and physical violence exposure: a sample from primary care patients BMC Public Health 2011 11 621 10.1186/1471-2458-11-621 21816070 PMC3160996

[B59] Liebschutz J Battaglia T Finley E Averbuch T Disclosing intimate partner violence to health care clinicians - what a difference the setting makes: a qualitative study BMC Public Health 2008 8 229 10.1186/1471-2458-8-229 18601725 PMC2474863

